# Physiological Candles

**Published:** 1889-11-23

**Authors:** 


### The Doctor's Pulpit.

### PHYSIOLOGICAL CANDLES.

### "Wonder" or "Form."

It is considered a sign of grace by the apostles of "good form" not to wonder at anything. Rightly understood, the absence of wonder is not a mark of grace, but of stupidity. Children and uneducated persons wonder at what they do not understand. They wonder for a moment, and then forget. Educated persons wonder at what they do understand, and their wonder is deep and permanent. Many people think that men of science have mastered the whole of every region of knowledge, and that the scientific leaders of the present time, like Alexander, are in despair for want of new worlds to conquer. But the real truth is that the conquests of science are in their infancy. From a scientific point of view the actual life and doings of the great mass of mankind are purely and entirely barbaric. Take, for example, the science of human physiology: What does the average man in the street know about it? And yet that average man is himself a walking physiological laboratory, in whom all the wonderful processes of physiology are being carried on all the day long. "Physiology is for doctors," you say; "and if I pay my medical man to understand physiology for me, why should I undertake the labour of understanding it myself?" But the very fact that the average man thinks the doctor, and the doctor alone, the natural and proper person to understand human physiology, is proof positive that the average man is so utterly destitute of science as not to have the slightest comprehension even of the value of it. That is indeed the very point we would be at. The actual, practical world of the nineteenth century, so far from being in any sense scientific, does not know the difference between the gold and the bronze of science. It has not got beyond the alphabet—it cannot spell words of one syllable.

What could be more extraordinary and even humiliating than the little episode at Newcastle-on-Tyne the other day? A workman quoted from Herbert Spencer a philosophical principle which is nothing at all if not practical; and Mr. Spencer comes forward to explain that his principles, though very sound in themselves, are not always such as can be applied in practice by the men of the present generation. They may be very suitable for future ages or for distant planets, but the men of to-day, he seems to think, are no more fit to put his philosophy into practice than a gorilla is to play the Dead March in "Saul." But if Mr. Herbert Spencer be right, and most people think he is, what an absurd thing it is to consider that the present is in any wide sense a scientific age! The truth is that we have a few eminent men of science, as Greece had before the Christian era, but it is very doubtful whether the mass of both educated and uneducated modern people are any more scientific in temper and practice than was Greece in the days of Aristotle.

Is there any use in putting the matter in this light? Every use! Large numbers of men, and especially young men, and still more especially young men of academic training, get a smattering of scientific facts, and a knowledge of a few leading hypotheses; and they straightway conclude that they occupy a scientific platform, and that they are looking at everything in life with scientific eyes. Whereas those very young men of the schools in most cases know no more of real science than the labels with which the facts they think they understand are marked. But because they think they know, therefore they never try to learn, exactly as a drunken Scotchman at Wick, who dreams he is in London, never thinks of taking the train and traversing the six hundred miles that separate him from the metropolis. Not only so, but the man who thinks his little smattering of science represents practically all that is to be known looks upon science as "played out," just as he looks upon his fast life and amusements as played out when he is tired of them.

Any single department of human physiology is an admirable candle for revealing to the average man how little he knows of science in an actual and practical sense. In an ordinary human being there are several more or less distinct and separate "systems," as they are called. There is the alimentary system, very complex, very wonderful, and very little understood, even by physiologists. There is the reproductive system, still more wonderful and still less understood; there is the circulatory system, which may be viewed separately; and the nervous system, which may also be considered in its entirety. Now all these separate systems are full of wonders, of astonishments. Not of wonders and astonishments to the child or the average man who sees only the outside, but to the man of science, to the man who knows most about them. Take, for example, the internal ear, which represents but a small part of the entire nervous system! To say nothing of the construction of the bony and muscular parts, which in themselves are sufficiently wonderful, what a miracle—a miracle that is in the classical sense of the word—what a "sign" is the nervous mechanism of the internal ear. The "organ of Corti," so well known to physiologists, is infinitely more wonderful in itself than the steamship, the telegraph, the telephone, or the electric light. That that small organ, so minute in its details as only to become visible under the higher powers of the microscope; that it should possess such rare endowments, not merely of hearing, but of discriminating ten thousand different sounds; not merely of discriminating, but of passing the most complex ideas on to the consciousness, and of communicating to the soul of the musician an overwhelming delight which language cannot even attempt to describe, would be incredible if it were not an actual fact. Is that nerve expansion—with its functions so wonderful that no man of science would believe them to be possible if they were not demonstrated and incontestible—is it so wonderful, and yet shall it be considered "bad form" to wonder at it? Tried by the standard of intelligent science, the man who chatters about "good form," and considers the expression of wonder an evidence of "bad form," is a lunatic, and ought forthwith to be sent to Virginia Water.

Wonder, in the sense of curiosity, is often the beginning of knowledge; wonder, in the sense of worship, is often the end of it. And why should a man not worship? Does it injure the child or check his developing intellect if he has a father he can honour and revere, and trust as he trusts the sun to rise or the night to fall? Does it not expand, and strengthen, and clear his intellect more than any educational method known to the schools? Exactly so does increasing knowledge of the more hidden facts of science lead to increasing wonder and worship; and the wonder and worship clear the eye of the grown man, strengthen and expand his intellect, and turn the barbarian and the vain fool into creatures of reason, of patience, of discretion, and of justice.

Persons who think much of "form," and they are many, exercise vast influence and authority in an age of wealth and leisure. Form has its considerable value when it consists in good behaviour inspired by good sense and good feeling. But when it is constituted by mere arbitrary custom and observance which have no foundation in either good sense or good feeling it is manifestly ridiculous, and deserving only of contempt. Religion, it might have been thought, would have been potent enough to liberate the minds of men, and even of women, from the tyranny of a slavish devotion to mere form. But if reasonable religion is beyond the comprehension of the idle wealthy, science, which is less lofty, may, perhaps, secure hearing and attention at their hands. One thing is certain, that any kind of "form" which tends to repress worthy and natural emotions tends in the same degree to dwarf the intellect and to impair the nobility of the character. To such "form" science is an uncompromising enemy. Wonder is natural to all unsophisticated men and women; and the greater the knowledge a man acquires both of objects external to himself, and of the operations of his own mind, the more will he be expanded and exalted by a never-ceasing wonder, and an increasing desire to press forward into the kingdom of knowledge which ever widens before him. To attempt to subdue the faculty of wonder in man or child is to inflict an injury which can never be repaired. Among all the teachings of physiological science none is plainer than this, that man is a living wonder; and any man who can see nothing wonderful in himself or his fellows is but little removed from those comparatively mindless creatures we have all agreed to call the "lower animals."

